# Prophylactic Antibiotics After Cleft Lip and Palate Reconstruction: A Review From a Global Health Perspective

**DOI:** 10.7759/cureus.36371

**Published:** 2023-03-19

**Authors:** Ellen M Piccillo, Cameron J Farsar, David M Holmes

**Affiliations:** 1 Otolaryngology, University at Buffalo Jacobs School of Medicine and Biomedical Sciences, Buffalo, USA; 2 Surgery, University of Connecticut Health, Farmington, USA; 3 Family Medicine, University at Buffalo Jacobs School of Medicine and Biomedical Sciences, Buffalo, USA

**Keywords:** surgical site infection, low-income countries, palatal fistula, postoperative management, cleft palate, cleft lip

## Abstract

Orofacial clefts are common congenital deformities. Global initiatives have increased access to cleft care and reconstruction surgeries for cleft lip with or without cleft palate (CL/P), but there is no consensus on the use of postoperative prophylactic antibiotics. We conducted a narrative review using PubMed on the use of postoperative prophylactic antibiotics in CL/P surgery. A search of PubMed identified 30 potentially relevant articles, of which 15 were reviewed. There was no consensus among surgeons on prescribing patterns, but there was limited evidence that postoperative antibiotics reduce palatal fistulas. Notably, microbiological screening is not used to guide the choice of antimicrobial or to predict postoperative complications. Based on limited available data, we cannot make any strong evidence-based recommendations on prescribing postoperative antibiotics; however, we recommend that each cleft surgeon performing these procedures in lower-income countries without access to tertiary care centers consider the cost-benefit analysis of prescribing antimicrobials postoperatively, without antimicrobial screening, which showed no benefit.

## Introduction and background

Orofacial clefts are one of the most frequently encountered congenital malformations worldwide, with an incidence of 1/1,000 for cleft lip with or without a cleft palate (CL/P) and 1/2,500 for cleft palate alone (CPO) [1]. These deformities result from the failed fusion of the lip and palate during fetal development [2]. Children born with this condition require multidisciplinary support to address airway management and feeding difficulties as well as psychosocial support [3]. Orofacial clefts are corrected by staged surgical interventions, with cleft lips repaired during the first year of life and cleft palates repaired before 18 months of age [4]. However, in low- to middle-income countries where access to appropriate healthcare is limited, single-stage cleft lip and palate repair is possible [5].

Because of the high number of cases of CL/P and CPO worldwide in combination with global health disparities, there have been many initiatives to increase the availability of cleft care and surgery. The goals are to repair the defect and minimize postoperative complications such as surgical site infection (SSI) and palatal fistula. Cleft lip and palate repair occurs in the oral cavity and is considered a clean-contaminated wound, justifying the use of postoperative antibiotics [6]. However, there are no formal guidelines regarding the use of postoperative prophylactic antibiotics in cleft lip or palate surgery [7].

This article reviews the current literature found using PubMed on the use of postoperative prophylactic antibiotics in cleft lip and palate surgery and examines their usefulness. These results are interpreted from a global health perspective, which comprises challenges that may differ from those of higher-income countries. To our knowledge, this is the first article of its kind with this point of view.

## Review

**Figure 1:**
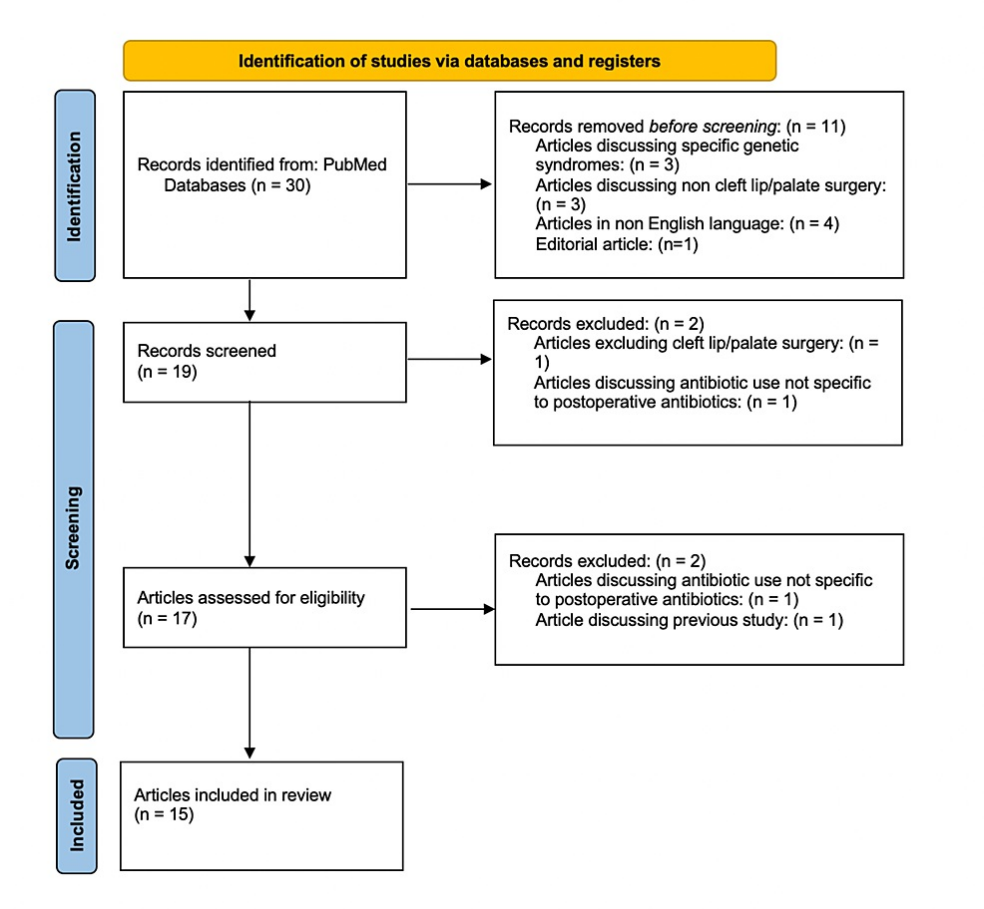
Flow chart showing screening process of literature review on PubMed Figure adapted from Page MJ, McKenzie JE, Bossuyt PM, et al.: The PRISMA 2020 statement: an updated guideline for reporting systematic reviews. BMJ. 2021, 372:n71. 10.1136/bmj.n71.

We conducted a narrowed literature review with the aim to examine articles discussing the use of antibiotics in surgical repair of CL/P and CPO specific to the postoperative period. A literature search of PubMed was conducted in June 2022 using the following terms: ((("prophylactic"[Title/Abstract] OR "prophylaxis"[Title/Abstract]) AND ("antimicrobial"[Title/Abstract] OR "antibiotic"[Title/Abstract] OR "antibiotics"[Title/Abstract])) OR "antibiotic prophylaxis"[MeSH Terms]) AND ("cleft lip"[MeSH Terms] OR "cleft palate"[MeSH Terms] OR "cleft lip"[Title/Abstract] OR "cleft palate"[Title/Abstract] OR "orofacial cleft"[Title/Abstract] OR "orofacial clefts"[Title/Abstract] OR "palatoplasty"[Title/Abstract] OR "cheiloplasty"[Title/Abstract]). Articles discussing specific genetic syndromes resulting in cleft lip or palate were excluded. Articles discussing antibiotic use but not specific to postoperative antibiotic use were excluded. Articles not focused specifically on cleft lip and palate surgery were excluded. There were no limitations on the year the study took place, the age of participants, or the location of the study. Articles were limited to the English language. The format of our literature review can be seen in Figure 1.

Our search yielded 30 articles, of which 15 articles were excluded according to our criteria; thus, 15 articles were reviewed. We found one article discussing consensus statements for the use of antibiotics in various types of plastic surgery [8]. This article discusses a meta-analysis performed by Aryan et al. in order to establish a consensus statement from the American Association of Plastic Surgeons. Regarding clean-contaminated head and neck procedures, the American Association of Plastic Surgeons acknowledges minimal evidence that antibiotic prophylaxis reduces SSI but recommends a one-time preoperative dose of antibiotics and no postoperative use. Rigotti et al. used the Research and Development Corporation and University of California-Los Angeles appropriateness method to develop consensus statements on the use of prophylactic antibiotic use in CL/P surgery [9]. They recommended that neonates or pediatric patients undergoing cleft operations receive a perioperative dose of ampicillin/sulbactam of 50 mg/kg. These recommendations were based mostly on practice patterns of cleft surgeons and heightened concern for complications such as palatal fistula and further surgery. Their recommendations did not include continuation of the antibiotic postoperatively.

Four studies examined the practice patterns of physicians and whether they used prophylactic antibiotics (Table 1) [10-13]. There was no consensus on the use of postoperative antibiotics. Krizek et al. found that only a small percentage of surveyed plastic surgeons always use antibiotics during cleft lip and palate surgeries [10]. When surveying these same surgeons about the timing of antibiotic use, only 23% of surgeons use prophylactic antibiotics after an operation in all of their plastic surgery procedures. Smyth and Knepil found that half of the surgeons who used antibiotics preoperatively for an isolated cleft lip reconstruction continued to use antibiotics postoperatively [11]. Rottgers et al. found that 23% of the 115 surgeons surveyed continued antibiotics for patients 24 hours after the primary palatoplasty procedure, with roughly half of them continuing use for up to 10 days [13]. Preidl et al. found that 40.3% of the surveyed surgical departments have a standardized postoperative antibiotic regimen [12].

**Table 1:** Results of literature review with regard to practice patterns of prescribing postoperative prophylactic antibiotics *Only discussed length of treatment for isolated cleft palate surgery.

Source	Percentage of surgeons using postoperative prophylactic antibiotics for CL/P surgery	Most common antimicrobial agent	Most common length of treatment (days)
Smyth and Knepil 2008 [11]	~50%	Amoxicillin/clavulanic acid	5*
Preidl et al. 2020 [12]	40.3%	Penicillins	3.4
Rottgers et al. 2016 [13]	63.6%	Cefazolin/cefalexin	1

Schonmeyr et al. conducted a study during a cleft lip mission trip to India and implemented an educational program targeted at improving postoperative care and decreasing complications [14]. A binary logistic regression revealed that the use of postoperative prophylactic antibiotics was not related to the reductions in wound dehiscence and wound infections. A randomized controlled trial published in 2015 evaluated the use of postoperative antibiotics in cleft palate surgery and found that the use of oral amoxicillin (50 mg/kg body weight/day) for five days reduced the rate of fistulas from 17.1% to 10.7%. However, this trial was included in a systematic review published in 2021 that concluded that the evidence for reduced risk of SSI with postoperative antibiotics was lacking [7,15]. Additionally, a narrative review in 2020 did not find a statistically significant reduction in complications, such as fistula, with the use of postoperative antibiotics [16]. A retrospective study by Jodeh et al. demonstrated a higher prevalence of fistulas following palatoplasty in those receiving postoperative antibiotics than in those receiving preoperative or no antibiotics [17]. The odds for an oronasal fistula requiring repair were initially significantly higher when postoperative antibiotics were used; however, the relationship was no longer significant when patients undergoing concurrent oronasal fistula repair and alveolar bone grafting were excluded from the analysis.

A 2016 quality improvement study at Boston Children’s Hospital examined the cost of prophylactic antibiotics after cleft lip surgery [18]. The group concluded that the cost of administering this treatment to patients is $3,000-$4,000 per year. They determined that foregoing the use of antibiotics after cleft lip surgery could save the plastic surgery department between $18,000 and $22,000 per year. 

We reviewed six studies discussing preoperative microbiological screening (Table 2) [19-24]. In all cases, samples were collected before the administration of intraoperative antibiotics. Our findings revealed no evidence supporting any proposed benefits of preoperative microbiological screening of individuals undergoing CL/P surgery. Thomas et al. found few similarities between microbiological samples taken preoperatively or intraoperatively and had no relationship with palatal fistula development [21]. Ranzer et al. systematically reviewed Thomas et al. and stated similar findings [19]. Adeyemo et al. found that bacteremia persisted in patients undergoing CL/P surgery when looking at venous blood samples taken 15 minutes after the final suture was placed and recommended the inclusion of prophylactic antibiotics as a standard of care [20].

**Table 2:** Results of literature review with regard to use of microbiological screening in predicting postoperative complications or guiding antibiotic use *This study listed number of samples growing each specific microorganism and did not specify whether a single sample grew one or more organism. **Cohen’s unweighted kappa.

Source	Sample source	Positive sample definition	Time of sample collection	Percentage of positive samples	Relationship of microbiology to postoperative complications	Relationship of preoperative microbiology to postoperative microbiology
Adeyemo et al. 2013 [20]	Venous blood	Positive microorganism growth at 14 days	Preoperatively, 1 minute after placement of last suture, 15 minutes after placement of last suture	38.1%	N/A	Persistent bacteremia in 35% of positive cultures at 1 minute
Chuo and Timmons 2005 [22]	Nose, throat and ear	Microbiology culture growing *Staphylococcus aureus* or β-hemolytic strep	Preoperatively	27.9%	N/A	N/A
Narinesingh et al. 2011 [23]	Nose and throat	Description of microbiology report 1+ to 3+	Preoperatively	53%*	50% of cases positive for *Moraxella* *catarrhalis* samples resulted in palatal fistulas (p < 0.05)	N/A
Rennie et al. 2009 [24]	Throat	Growth of microorganisms on culture	Preoperatively	15%	30% of cases with positive sample developed palatal fistula (p = 0.208)	N/A
Thomas et al. 2012 [21]	Nose and throat	Positive microorganism growth at 48 hours	Preoperatively	42.4%	N/A	κ = 0.196**
			Intraoperatively	62%		

The field of global cleft lip and palate repair has evolved, with many organizations taking trips and establishing cleft centers in lower-income countries. These regions have limited access to care and resources, and patients often have less knowledge of their condition. Massenburg et al. surveyed healthcare workers partnering with Smile Train and found that the most commonly reported barriers to cleft care were patient travel costs, lack of patient awareness, and lack of financial support [25]. In Nigeria, a high rate of the patient dropout was reported, and patients subsequently did not receive cleft palate repair [26]. This was attributed to a lack of patient/guardian understanding of the necessity of care.

Palatal fistulas as a sequela of infection after cleft surgery can present anytime between one and 78 months (mean, ~9 months) after surgery [27]. Our search revealed some, though limited, data supporting the use of postoperative antibiotics to prevent fistula development. Additionally, our data showed that bacteremia can persist after these procedures. The risk for such complications may be greater for patients in areas without the appropriate care. For example, many patients in lower-income countries do not have the same access to hygiene care or clean water as higher-income countries, resulting in increased exposure of the wound to infectious agents. Moreover, patients that develop an SSI or palatal fistula may not receive prompt treatment because of limited transportation availability and/or cleft center accessibility and thus present with a more complicated case. In some cases, patients treated by surgeons on mission trips where there is no follow-up cleft care may not have access to treatment again until the surgery team returns. Depending on the specific surgical team, the time frame of the return could vary widely. 

The Surgical Infection Society justifies the use of prophylactic antibiotics in cases where the morbidity associated with surgical infection is greater than the morbidity associated with prophylactic antibiotic use [6]. For example, the risk of infection with cardiac surgery is low; however, the consequences of an SSI can be dire and could result in the need for a second cardiac operation to address the infection. We argue that prophylactic antibiotics are similarly justified for patients receiving cleft surgery in lower-income countries due to the lack of access to routine follow-up or tertiary care centers equipped to identify and correct SSI or palatal fistulas.

Based on the data in the reviewed studies, we cannot make any strong data-driven recommendations on antimicrobials after cleft surgery. Only one of our reviewed studies showed a significant decrease in palatal fistulas with the use of postoperative antibiotics. However, we advise that cleft surgeons practicing in resource-poor areas of the world with challenges as previously discussed consider the cost-benefit analysis of implementing a prophylactic antibiotic regimen without microbiological screening, which was not shown to provide any benefit. We are cognizant of antibiotic stewardship and microbial resistance as well as potential adverse effects of antibiotics, such as a type I hypersensitivity reaction. Notably, Schonmeyr et al. showed that the implementation of a patient education program and standardization of postoperative care among surgeons and pediatricians reduced postoperative antibiotic use from 46% to 4% with a concurrent reduction of postoperative complications [14]. This could serve as a model for an alternative to postoperative antibiotic use while also protecting vulnerable patients from the potentially life-threatening complications of their surgeries. More research into the subject needs to be performed to establish high-quality, evidence-based guidelines to standardize care across the globe.

The small amount of available literature on the use of prophylactic antibiotics after CL/P and CPO reconstructions is a major limitation of this study. Only one randomized controlled study from a single institution was identified. Additionally, although our review focused on the global health perspective, four articles that were not written in English were excluded, thereby introducing a potential bias. Many articles discussed were performed in high-income countries, further contributing to this potential bias.

## Conclusions

Orofacial clefts are a common congenital malformation that requires surgical correction and multidisciplinary support. There is no consensus on the use of postoperative prophylactic antibiotics for these surgeries to prevent complications such as palatal fistula and SSI. However, our review of 15 studies concluded some evidence that postoperative prophylactic antibiotics reduce postoperative complications such as palatal fistula. Based on limited available data, we cannot make any recommendations on prophylactic antibiotic use; however, we advise cleft surgeons performing cleft lip and palate surgery in lower-income countries to consider prescribing antimicrobials postoperatively, without the use of antimicrobial screening which showed no benefit.
